# Fluconazole and Lipopeptide Surfactin Interplay During *Candida albicans* Plasma Membrane and Cell Wall Remodeling Increases Fungal Immune System Exposure

**DOI:** 10.3390/pharmaceutics12040314

**Published:** 2020-04-01

**Authors:** Jakub Suchodolski, Daria Derkacz, Jakub Muraszko, Jarosław J. Panek, Aneta Jezierska, Marcin Łukaszewicz, Anna Krasowska

**Affiliations:** 1 Faculty of Biotechnology, University of Wroclaw, 50-383 Wroclaw, Poland; jakub.suchodolski@uwr.edu.pl (J.S.); daria.derkacz@uwr.edu.pl (D.D.); jakub.muraszko@uwr.edu.pl (J.M.); marcin.lukaszewicz@uwr.edu.pl (M.L.); 2 Faculty of Chemistry, University of Wroclaw, 50-383 Wroclaw, Poland; jarek@elrond.chem.uni.wroc.pl (J.J.P.); anetka@elrond.chem.uni.wroc.pl (A.J.)

**Keywords:** *Candida albicans*, cell wall, *β*-glucan, lipopeptide, surfactin, fluconazole

## Abstract

Recognizing the *β*-glucan component of the *Candida albicans* cell wall is a necessary step involved in host immune system recognition. Compounds that result in exposed *β*-glucan recognizable to the immune system could be valuable antifungal drugs. Antifungal development is especially important because fungi are becoming increasingly drug resistant. This study demonstrates that lipopeptide, surfactin, unmasks *β*-glucan when the *C. albicans* cells lack ergosterol. This observation also holds when ergosterol is depleted by fluconazole. Surfactin does not enhance the effects of local chitin accumulation in the presence of fluconazole. Expression of the *CHS3* gene, encoding a gene product resulting in 80% of cellular chitin, is downregulated. *C. albicans* exposure to fluconazole changes the composition and structure of the fungal plasma membrane. At the same time, the fungal cell wall is altered and remodeled in a way that makes the fungi susceptible to surfactin. In silico studies show that surfactin can form a complex with *β*-glucan. Surfactin forms a less stable complex with chitin, which in combination with lowering chitin synthesis, could be a second anti-fungal mechanism of action of this lipopeptide.

## 1. Introduction

*Candida albicans* is a component of the resident microflora present in the human digestive tract, skin, and mucosal membranes. It is an opportunistic human pathogen, meaning infections normally occur only in immunocompromised individuals. Systemic *C. albicans* infections can occur after invasive medical treatments like injections and surgeries. The mortality rate is near 40% for systemic infections [1]. One contributing factor to the mortality rate is the ability of *C. albicans* to effectively hide from the immune system due to its normal environmental niche as a member of the resident microbiota [2,3]. Another contributing factor to the mortality rate is that safe and effective treatment options are limited. Since the discovery of the first antifungal azole compound in 1944 [4], only a few new drug classes have been discovered [5]. In addition, all antifungal drug classes have a minimal number of targets. Azoles like fluconazole and itraconazole belong to a class of heterocyclic compounds containing a nitrogen atom and at least one other non-carbon atom. These compounds alter the plasma membrane (PM) by inhibiting sterol synthesis [6,7]. Polyenes like amphotericin B and nystatin also affect the PM by binding to ergosterol [8,9]. The newest class of antifungal compounds called echinocandins includes caspofungin and micafungin. Echinocandins are lipopeptides containing large cyclic (hexa)peptides that affect the cell wall by inhibiting *β*-glucan synthase [10,11]. Thus, many antifungal drug classes primarily target the PM and the cell wall.

The PM usually forms a boundary between the cell and its external environment. However, the *C. albicans* PM has no direct contact with the external environment. The outer surface of the *C. albicans* cell that has direct contact with the environment and the host immune system is called the cell wall [3]. The *C. albicans* cell wall is 90% carbohydrates and 10% proteins [12]. The fibrillar cell wall outer layer is mainly composed of mannoproteins. The carbohydrate components of the inner wall are *β*-glucans and chitin. This is the closest layer to the PM [13].

The cell wall serves many necessary cellular functions [14]. The cell wall maintains consistent cellular morphology and interacts with the external environment. Despite the cell wall’s function to maintain cellular shape and establish mechanical resistance, the fungal cell wall is exceptionally flexible and dynamic. The cell wall structure allows for rapid changes in cellular volume, cellular division, hyphae and pseudohyphae formation, and extracellular vesicle secretion. Cell wall remodeling affects fungal-host interactions by exposing major cell wall-associated proteins that are then recognized by innate immune cells. Cell wall remodeling depends on interplay between the PM and the cell wall. This interplay is a highly regulated process. Only a few studies describe interactions between the PM and cell wall. Drugs that target the PM must first interact with the cell wall in order to reach the PM. When these drugs reach the cell wall, they can possibly adhere, accumulate, and undergo structural and/or chemical modifications like changes in hydrophobicity [15]. After a drug has penetrated the cell wall and altered the PM, this process can, in turn, affect cell wall structure. Previous research has shown that exposing *C. albicans* to azoles, which interfere with sterol synthesis, affects chitin biosynthesis and deposition [16].

Similarly, when *C. albicans* is exposed to echinocandins, *C. albicans* upregulates chitin synthesis, reducing drug efficacy [17]. Cell wall structural changes have been observed in response to environmental stress and other changes. Masking immune-stimulating cell wall components facilitates immune evasion. However, cell wall remodeling may expose these components and lead to recognition of *C. albicans* by the immune system [18,19,20]. As it is difficult to discover totally new and efficient antifungal compounds, one prominent drug development strategy is to look for compounds that are synergistically active with existing therapeutic compounds [21].

Lipopeptides are promising compounds with the potential for synergy with existing antifungals [22]. Echinocandins are a group of compounds with these properties. Lipopeptides are composed of a hydrophobic fatty acid residue and a hydrophilic amino acid chain. These moieties are often enclosed in a ring by a lactone bond. Due to the chemical diversity of the fatty acid residue and the peptide chain, this is very large group of compounds. The fatty acid residue can have different chain lengths, different types of branching, or the presence of hydroxyl groups. The peptide chain may contain a variety of amino acids in both L and D configurations. Lipopeptides are produced by many microorganisms, including microorganisms present in the microbiome with the potential to affect colonization by *C. albicans.* Besides having surfactant activity, different lipopeptides have shown to have antibacterial, antifungal, anti-adhesion, quorum sensing, or anticancer activities [23,24,25]. For example, the lipopeptides pseudofactin II and surfactin (SU) decrease *C. albicans* adhesion and hydrophobicity [15,25]. SU is produced by *Bacillus* species, including *B. subtilis* strains used in production of fermented foods like natto [26]. The health benefits of consuming fermented foods may be related to the presence of SU and its ability to act upon pathogenic microorganisms. SU may integrate into the PM and change its properties.

In this study, we investigated synergistic activity between SU and azoles. Azoles block ergosterol biosynthesis through inhibition of the 14 α-methyl sterol demethylase (P450 cytochrome) encoded by the *ERG11* gene. Strains containing a deletion in *ERG11* show similar phenotypes to cells treated with azoles. Azole treatment results in accumulation of lanosterol instead of ergosterol in the PM. Both azole treatment and *ERG11* deletion results in increased susceptibility of *C. albicans* to SU. These results show the strong synergistic activity of this biosurfactant with azoles. Modifying the PM affects cell wall shape and local accumulation of chitin. Changes in the cell wall lead to the unmasking of *C. albicans* chitin and *β*-glucan. Molecular dynamics studies show that SU can form intermolecular complexes with chitin and *β*-glucan.

## 2. Materials and Methods

### 2.1. Strains and Growth Conditions

*C. albicans* strains used in this study were CAF2-1 (genotype: *ura3Δ::imm434/URA3*, treated here as wild type, WT) [27], and KS028 (genotype: same as CAF2-1 but *erg11Δ::SAT1-FLIP/erg11Δ::FRT*) [28]. The medium used to culture strains was yeast extract-peptone-dextrose (YPD) medium (1% yeast extract, (manufacturer: BD; distributor: Diag-med; Warszawa, Poland); 2% peptone, (manufacturer: BD; distributor: Diag-med; Warszawa, Poland) and 2% dextrose, (manufacturer: Bioshop; distributor: Lab Empire; Rzeszów, Poland). Agar (manufacturer: Bioshop; distributor: Lab Empire; Rzeszów, Poland) in a final concentration of 2% was used for medium solidification. For specific experiments, cells were grown in 20 mL of YPD (28 °C; shaking: 120 rpm; starting A_600_ = 0.1; with or without fluconazole (FLC) (Sigma-Aldrich; Poznan, Poland), surfactin (SU) or the combination of both drugs added at t = 0 h) until they reached the stationary phase (24 h).

### 2.2. Determination of Azole-SU Synergy, Percentage of Viability and Fractional Inhibitory Concentration Indexes

To assess the effects of azoles (fluconazole, ketoconazole, itraconazole, miconazole or clotrimazole) (Sigma-Aldrich), SU or azole-SU combinations on *C. albicans* viability, we followed the protocol of Clinical and Laboratory Standards Institute (2008), 3rd ed. M27-A3 [29], with modifications described before [30,31]. Stock solutions of xenobiotics (alone or in combination) were serially diluted in YPD medium using 96-well sterile plates (Sarstedt; Nümbrecht, Germany). They were then inoculated with *C. albicans* suspensions (final A_600_ per well = 0.01) and prepared in fresh YPD medium from 24 h YPD cultures. After 24 h incubation at 28 °C, A_600_ was measured (ASYS UVM 340 Biogenet; Józefów, Poland). The percentage of viability (% viability) of CAF2-1 and KS028 was determined by normalizing A_600_ to experiments where the condition without the addition of xenobiotics was counted as 100% viability. The concentrations of xenobiotics (alone or in combination) were determined as either ≥50% or ≥90% growth inhibition. Fractional inhibitory concentration indexes (FICI) were calculated according to the formula:

$$FICI=\frac{SU\;in\;combination}{SU\;alone}+\frac{Azole\;in\;combination}{Azole\;alone}$$

### 2.3. Plasma Membrane (PM) Permeabilization

To assess the effects of SU on CAF2-1 and KS028 PM permeability we followed the propidium iodide (PI) (Bioshop) assay [32], with modifications. Briefly, CAF2-1 and KS028 were grown until stationary phase of growth (24 h), pelleted (4 k × g, 5 min) and resuspended in YPD of A_600_ = 0.1. Cells were exposed towards SU for 2 h at 28 °C, pelleted, washed twice with 0.9% saline (Stanlab; Lublin, Poland) (4 k × g, 5 min) and resuspended in 0.9% saline with 6 µM PI for 5 min. Then, cells were pelleted, washed twice with 0.9% saline (4 k × g, 5 min), concentrated and observed under a Zeiss Axio (Poznan, Poland) Imager A2 microscope equipped with a Zeiss Axiocam 503 (Poznan, Poland )mono microscope camera and a Zeiss HBO100 mercury lamp (Poznan, Poland). The percentage of permeabilisation was evaluated by counting PI positive cells out of at least 200 cells in three independent replicates for each experiment (*n* = 600).

### 2.4. Scanning Electron Microscope (SEM) Observations

SEM observations were performed to evaluate the effects of SU on CAF2-1 and KS028 ultrastructural cell surface changes according to Piętka-Ottlik et al. [33]. Briefly, *C. albicans* cells were pelleted, washed twice with 0.9% saline (4 k × g, 5 min), resuspended to A_600_ = 1 in 0.9% saline and fixed with 2.5% glutaraldehyde. The samples were subsequently treated with phosphate buffer in 2.5% glutaraldehyde, dehydrated by acetone washes and dried. SEM analyses were made with Hitachi S-3400N (Hitachi; Tokyo, Japan) equipped with a tungsten cathode (magnification 80–300.000x) at operation voltage of 15 keV. Micrographs have been acquired with a secondary electron detector (SE) and a backscattered electron detector (BSE).

### 2.5. Cell Wall Staining and Microscopic Observations

Total chitin visualization was performed by staining *C. albicans* cells with Calcofluor white (CFW) (Sigma-Aldrich), as described before [30], with modifications. Visualization of unmasked chitin was performed by staining *C. albicans* cells with wheat germ agglutinin conjugated with FITC (WGA-FITC) (Sigma-Aldrich), by modifying the protocol of Malavia et al. [34]. Unmasked β-glucans were stained according to the protocol of Wagener et al. [35]. In all cases, *C. albicans* cells were pelleted, washed twice with 0.9% saline (4 k × g, 5 min) and resuspended (to A_600_ = 1) in 0.9% saline with either 12.5 µM CFW, 50 µg/mL WGA-FITC or 5 µg/mL Fc–hDectin-1 (Invivogen). After 1h incubation cells were pelleted, washed twice with 0.9% saline (4 k × g, 5 min) and, in case of CFW and WGA-FITC staining, concentrated. Fc-hDectin-1 treated cells were incubated with 1:250 Alexa fluor 448-conjugated anti-human IgG Fc antibodies (Thermo fisher, Waltham, USA) for 1 h on ice. Afterwards, cells were pelleted, washed twice with 0.9% saline (4 k × g, 5 min) and concentrated. Preparations were observed under Zeiss Axio Imager (Oberkochen, Germany) A2 microscope equipped with a Zeiss Axiocam 503 mono microscope camera and a Zeiss HBO100 mercury lamp or in case of CFW-stained cells also under a Leica SP8 LSM confocal microscope.

### 2.6. Fluorescence-Activated Cell Sorting (FACS) Analyses

*C. albicans* cells were stained with WGA-FITC or Fc-hDectin-1 (Section 2.5) and fixed with 43.7% formaldehyde (Sigma-Aldrich; Poznan, Poland) for 15 min, pelleted, washed twice with 0.9% saline (4 k × g, 5 min) and resuspended in 0.9% saline. For each FACS analysis, cell suspensions were diluted from 1:20 to 1:100 in 0.9% saline, and 5000 events were collected on a Millipore Guava easyCyte 5HT flow cytometer (Merck; Burlington, USA). Gates were set around the cell population using the forward and side scatter channels. Fluorescence signal was obtained by using blue laser included in flow cytometer with a wavelength of 488 nm. Data was analyzed using InCyte software provided by Millipore (Merck; Burlington, USA).

### 2.7. RNA Preparation, Reverse Transcription and Quantitative Polymerase Chain Reaction (PCR)

Total RNA from *C. albicans* cells was isolated according to TOTAL RNA MINI kit provided by A&A Biotechnology (Gdynia, Poland). Cell pellet was incubated for 5 min in 50 °C with 800 μL Phenol-containing fenozol, followed by addition of 200 μL chloroform. Samples were centrifuged (10 k × g, 10 min), top fractions were collected and mixed with 250 μL of isopropanol. Subsequently, samples were transferred to minicolumns, and procedure was continued accordingly to manual provided by manufacturer. RNA was eluted with clean Milli-Q water.

To obtain cDNA for further analyses, isolation of RNA was followed by reverse transcription. Reaction was performed using High-Capacity cDNA Reverse Transcription Kit (ThermoFisher Scientific; Waltham, USA). RNA samples were mixed with buffer, reversed transcriptase, random primers and deoxyribonucleotides and incubated for 120 min in 37 °C. Samples were used as a matrix in quantitative PCR reaction.

Genes expression levels were measured by quantitative PCR, which was performed with iTaq Universal SYBR Green Supermix kit (BioRad; Warszawa, Poland). Reactions were run using Step One Real-Time PCR System (ThermoFisher Scientific). Calculations of gene expression levels were performed as previously described [36]. The following gene-specific primers were used: RDN18F (5′-AGAAACGGCTACCACATCCAA-3′), RDN18R (5′-GGGCCCTGTATCGTTATTTATTGT-3′), CHS3F (5′-GACGATCTTTTGTTGGTAATCC-3′), CHS3R (5′-CAAGTCATTTCATCTTCAAGACC-3′), CHS4F (5′-CACCGGGATCCAATATGC-3′), CHS4R (5′-GAAACAGTCTTTGTGACGAC-3′), GSC1F (5′-GGTGGTCTAATTAATCTTGATGG-3′), GSC1R (5′-GTCGGAATTTGCTGGTG-3′), GSL1F (5′- GCTGCTTTTGTACATTTTGC-3′), GSL1R (5′-GAAATTCGAAATGATTCTATGGC-3′) KRE9F (5′-CCATAATAAGATGTGTTGTTGCAG-3′), KRE9R (5′-TCCAAAGATTTCGGTGAGTC-3′).

### 2.8. Computational Methodology

The initial model of the SU was based on the *B. subtilis* SU with C14 aliphatic chain and the sequence ELLVDLL (amino acid configuration LLDLLDL), PDB ID: 2NPV [37]. The hexameric *N*-acetylglucosamine and β-D-glucose representing chitin and *β*-glucan respectively were drawn manually in linear arrangement using the Molden 4.9 program [38]. The molecular topologies and parameters were then prepared using Antechamber module of the Amber2015 suite of programs [39] and general-organic Amber force field GAFF [40] with AM1-BCC [41] atomic charges. Then, initial structures were prepared by placing the SU and hexasaccharide molecules in separation of approximately 4 Å, placing them in a cuboid box, neutralizing with randomly placed Na^+^ ions, and solvating the box with TIP3P water [42]. The noncovalent interaction cutoff was set to 10 Å, and the long-range electrostatics was treated with Particle Mesh Ewald [43] technique. Further simulations proceeded in the following manner: Initial steepest-descent minimization of 1000 steps was used to remove bad contacts. Then, constant-volume (NVT) thermalization at 300 K was carried out for 100 ps, followed by 400 ps of constant-pressure equilibration at T = 300 K, p = 1 atm. The final NVT production run lasted for 100 ns. All the molecular dynamics runs used time step of 1 fs and Andersen temperature coupling scheme with collision frequency of 1 ps [44]. Each of the two cases, SU-chitin and SU-*β*-glucan complexes, was run in triplicate so that the random generation of initial velocity distribution and thermostatting collisions allowed the three runs for each system to explore different parts of the conformational space. The post-production analysis and visualization were carried out with the tools of the Amber2015 suite of programs and with the Visual Molecular Dynamics VMD 1.9.3 program [45].

### 2.9. Statistical Analysis

Unless stated otherwise, data represent the means ± standard errors from at least 3 biological replicates. Statistical significance was determined using Student’s t-test (binomial, unpaired). Microscopic observations and FACS analyses were performed at least in 3 independent replicates, representatives were included in figures.

## 3. Results

### 3.1. C. albicans Strain Deficient in Erg11p Are More Susceptible to Surfactin Treatment

**Figure 1:**
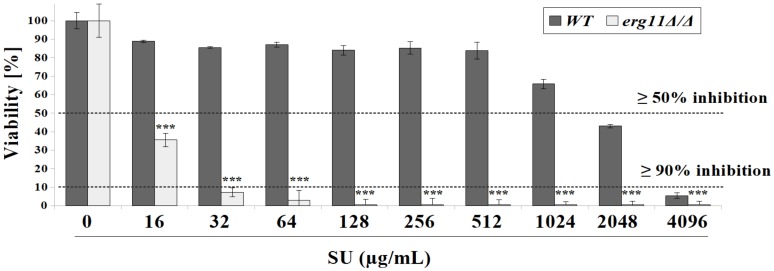
The percent viability of *C. albicans* CAF2-1 (wild type, WT) and KS028 (*erg11Δ/Δ*) strains after 24 h growth in yeast extract-peptone-dextrose (YPD) in the presence of surfactin (SU) (0–4096 µg/mL). Concentrations of surfactin (SU, µg/mL), leading to either ≥50 or ≥90% growth inhibition towards *C. albicans* CAF2-1 (WT) and KS028 (*erg11Δ/Δ*) strains were indicated by dashed lines and were equal to 2048 and 16 (≥50% inhibition) for WT or *erg11Δ/Δ*, respectively and 4096 and 32 (≥90% inhibition) for WT or *erg11Δ/Δ*, respectively. Statistical analyses were performed by comparing viability of the *erg11Δ/Δ* strain to the viability of the WT strain in correspondence with SU concentrations (means ±SD, *n* = 6) (*, P < 0.05; **, P < 0.01; ***, P < 0.001).

Surfactin (SU), like other lipopeptides, can form channels in the cell membrane. Formation of these channels constitutes antibacterial activity [46]. Previous studies performed by our group have shown that SU did not kill *C. albicans* strains at the concentrations used [15]. Recently, it was observed that a *C. albicans* KS028 mutant with a deletion in *ERG11* is 128-fold more sensitive to SU than the parental strain CAF2-1 (Figure 1). *C. albicans* KS028 (*erg11Δ/Δ*) growth was inhibited when exposed to SU at 16 and 32 µg/mL by 64.5% and 93%, respectively (Figure 1). The viability of the *C. albicans erg11Δ/Δ* mutant was 50% and 80% lower than that of the *C. albicans* wild type strain CAF2-1 depending on the SU concentration (Figure 1).

### 3.2. Surfactin Acts in Synergy with Azole Compounds to Inhibit C. albicans Viability

A *C. albicans* mutant without the ability to synthesize ergosterol is a model example of azole-treated yeast cells. Fluconazole is one of the most commonly used azoles for treatment of infection. *C. albicans*, however, has developed a high drug resistance to fluconazole through multiple underlying mechanisms [47,48]. One alternative strategy for treating candidiasis is to treat with compounds that are synergistically active with azoles in combination with azole treatment [49,50,51]. In order to investigate whether SU is synergistically active against *C. albicans* in combination with azoles, we tested a combination of this lipopeptide with the triazole compounds, fluconazole and itraconazole. In addition, we tested SU in combination with the imidazoles ketoconazole, clotrimazole, and miconazole.

The concentration leading to ≥90% growth inhibition of triazoles alone was below the level of detection for the *C. albicans* CAF2-1 (wild type, WT) strain. This value was over 256 for fluconazole and 8 µg/mL for itraconazole. At a concentration of 16 µg/mL, SU reduced the value leading to ≥50% growth inhibition for fluconazole 4-fold. A concentration of 32 µg/mL SU reduced the ≥50% growth inhibition activity for fluconazole 4-fold and itraconazole 8-fold. Both concentrations of SU did not change the ≥50% growth inhibition activity of imidazoles (Table 1). The ≥90% growth inhibition concentration values of ketoconazole and clotrimazole were 512 and 32-fold lower, respectively, in the presence of SU. At the concentrations 16 and 32 µg/mL, SU lowered the concentration leading to ≥90% growth inhibition of miconazole 64 and 128-fold (Table 1).

### 3.3. Surfactin Permeabilized the Plasma Membrane of C. albicans erg11Δ/Δ Mutant and C. albicans WT Strain after Treatment with Fluconazole

**Figure 2:**
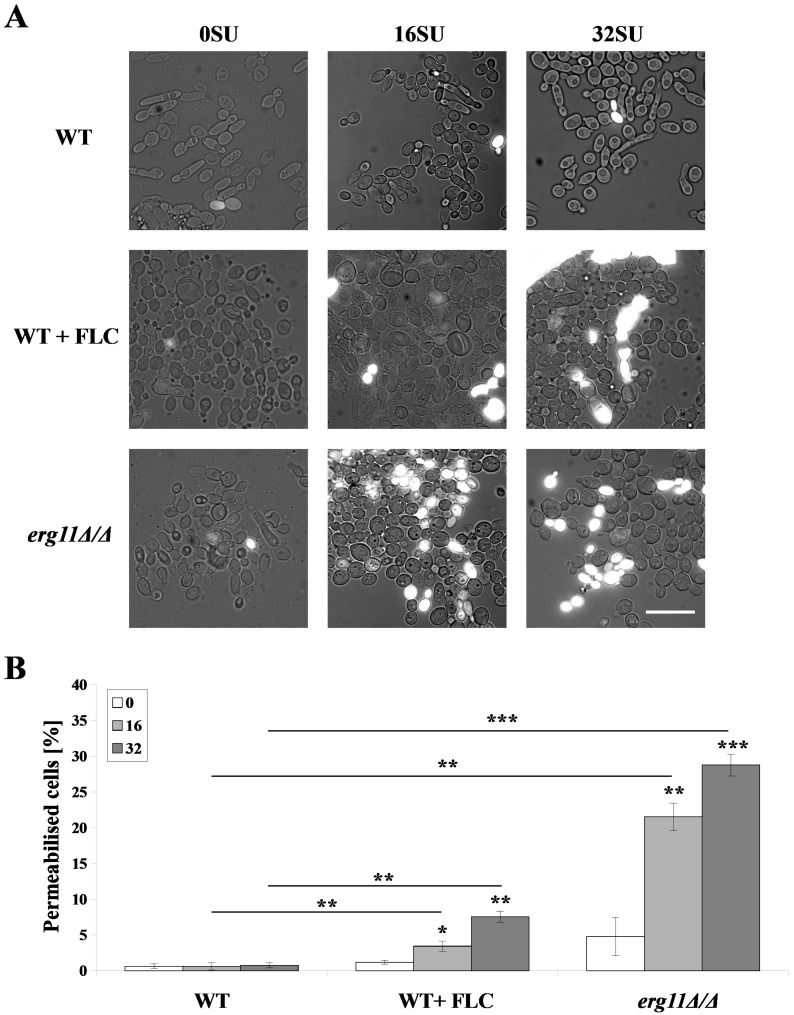
*C. albicans* CAF2-1 (WT) strains were grown in the presence of 1 µg/mL fluconazole pretreatment (WT + FLC). *C. albicans* KS028 (*erg11Δ/Δ*) strains were stained with propidium iodide (PI) after 2 h treatment with SU (0, 16 and 32 µg/mL). (**A**) Representative microscopic observations, scale bar = 20 µm; (**B**) Histograms of quantified % of permeabilized cells, means ± SD (*n* = 3), statistical analysis at each concentration was performed relative to control experiments without SU treatment (above bars) or between WT and WT + FLC or WT and *erg11∆/∆* strains (above lines) (*, P < 0.05; **, P < 0.01; ***, P < 0.001).

Changes in the PM structure of *C. albicans*, caused by various external factors, lead to PM permeabilization, ion leakage and cell death [52]. Sterols are the most abundant membrane components that influence membrane properties like tensile properties, phase separation properties, or liquid-ordered phase properties of the membrane [53,54]. Dupont et al. [52] proved that *Saccharomyces cerevisiae* mutants with alterations in the ergosterol biosynthetic pathway have permeabilized PMs. As a result, these cells were more sensitive to dehydration. We investigated whether this effect is due to reduction in the amount of ergosterol or the complete removal of ergosterol in *C. albicans* cells. We investigated which of these factors causes PM permeabilization and whether interaction with SU intensifies the process of PM permeabilization. We treated *C. albicans* WT and *erg11Δ/Δ* strains with 16 and 32 µg/mL of SU (Figure 2). Without SU treatment, the percent of permeabilized WT cells was less than 1%. A small increase in this percentage was observed after treatment with 1 µg/mL fluconazole. *C. albicans erg11Δ/Δ* cells not treated with SU were permeabilized in 4.8 ± 2.7%. The treatment with 16 and 32 µg/mL SU, respectively, resulted in 21.6 ± 1.9 and 28.8 ± 1.5% permeabilization of *erg11Δ/Δ* cells (Figure 2B). Treatment with SU and fluconazole increased the amount of permeabilized cells to 3.4 and 7.5%, depending on SU concentration (Figure 2B).

### 3.4. Ergosterol Depletion and Surfactin Treatment Cause Changes in Cell Shape and Local Accumulation of Chitin in the Cell Wall of C. albicans

**Figure 3:**
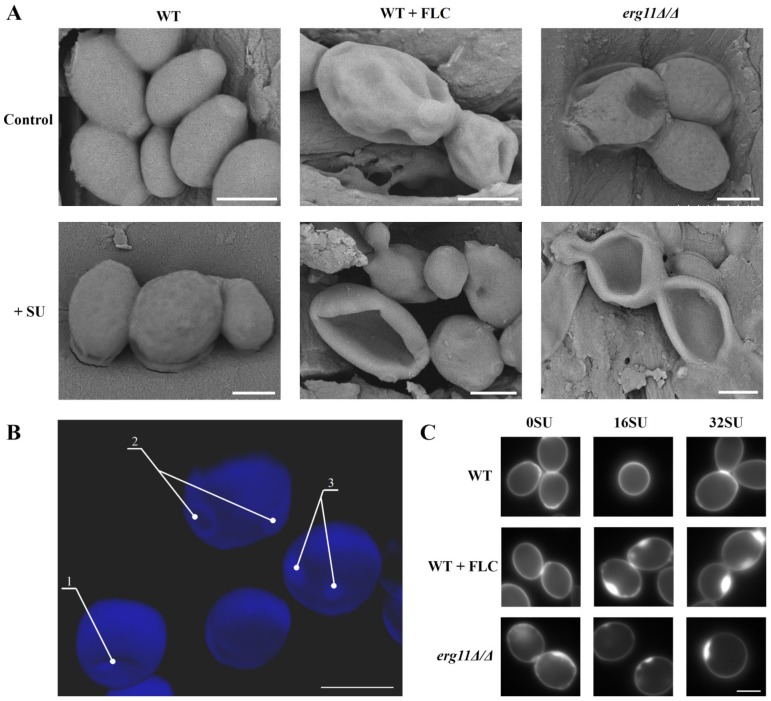
Scanning electron microscope (SEM) images of *C. albicans* CAF2-1 (WT) and KS028 (*erg11Δ/Δ)* strains. ((**A**)—upper panel) images of *C. albicans* cells untreated (control) or treated with 1 µg/mL fluconazole. ((**A**)—lower panel) images of *C. albicans* cells treated with 32 µg/mL SU, (**B**) Confocal microscope z-stack projection showing *C. albicans* KS028 (*erg11Δ/Δ*) chitin staining by calcofluor white dye (CFW); 1—surface dimples, 2—septa, 3—localized chitin accumulations. Scale bars in all images are equal to 2.5 µm. (**C**) *C. albicans* chitin was stained with calcofluor white (CFW) in *C. albicans* CAF2-1 (WT) untreated and treated with 16 or 32 µg/mL SU. The *C. albicans* CAF2-1 (WT) strain was simultaneously treated with 0.5 µg/mL fluconazole and 16 or 32 µg/mL SU. *C. albicans* KS028 (*erg11Δ/Δ*) was also treated under the same conditions. Images are representative of at least three independent experiments. Scale bars are equal to 2 µm.

Recently, Madhavan et al. [55] published results showing that fluconazole and voriconazole change the shape of *Candida glabrata, Candida parapsilosis* and *Candida rugosa* cells. Among other changes, these compounds cause dimples on the cell surface. We decided to check if changes in the amount of ergosterol in the *C. albicans* PM in combination with SU treatment lead to changes in cell shape. SEM images of *C. albicans* cells treated with fluconazole or SU, as well as depletion of ergosterol, revealed ultrastructural changes. *C. albicans* CAF2-1 cells with normal ergosterol levels are ovular with smooth surfaces. SU causes only a light wrinkled surface (Figure 3A, left panel). Fluconazole causes deformation of the cell surface and the formation of dimples which are deepened by SU treatment (Figure 3A, middle panel). The *C. albicans erg11Δ/Δ* mutant unable to produce ergosterol showed a similar shape as the cells treated with 1 µg/mL fluconazole (Figure 3A, right panel). In addition to SEM images, confocal microscope images were acquired of cells in the mutant strain unable to produce ergosterol with chitin-stained calcofluor white (CFW) (Figure 3B). This image showed localized accumulation of chitin. Dimples in cell shape and chitin accumulation was observed in locations other than the scars of buds.

In *C. albicans*, inhibition of *β*-glucan by caspofungin results in a compensatory increase in chitin synthesis. This effect is a defensive mechanism that makes the fungus resistant to caspofungin [14,56]. We observed accumulation of chitin in the *erg11Δ/Δ* mutant, suggesting a possible *C. albicans* defense mechanism to compensate for the lack of ergosterol. We investigated whether treatment of the WT strain with fluconazole leads to changes in the distribution of chitin in the cell wall. In addition, we investigated whether treatment with SU enhances any observed effects. Cell wall staining with calcofluor white showed even distribution of chitin in *C. albicans* CAF 2-1 cells. Chitin distribution did not change after SU treatment (Figure 3C). The same effect was observed with 0.5 µg/mL fluconazole treatment (Figure 3C). During concurrent fluconazole and SU treatment of *C. albicans* CAF2-1, chitin accumulation was observed (Figure 3C). The same effect was observed in the mutant unable to produce ergosterol (Figure 3C).

### 3.5. Surfactin and Fluconazole Cause Unmasking of Chitin and β-glucan in the Cell Wall of C. albicans

Chitin and *β*-glucan are two key fungal cell wall pathogen-associated molecular patterns (PAMPs) which are recognized by pattern recognition receptors (PRRs) expressed on the surfaces of innate immune cells [57,58]. The fungal cell wall is remodeled upon recognition of key host-derived environmental signals [59] or changes in carbon sources and pH [60,61]. Wheeler and Fink [62] found that *β*-glucan in the cell wall of *C. albicans* is unmasked by caspofungin. The results we have obtained indicate changes in the *C. albicans* cell wall are due to depletion or lack of ergosterol and enhancement of this effect by SU. As a result, we investigated whether there are changes in the masking of chitin and *β*-glucan under the above conditions.

**Figure 4:**
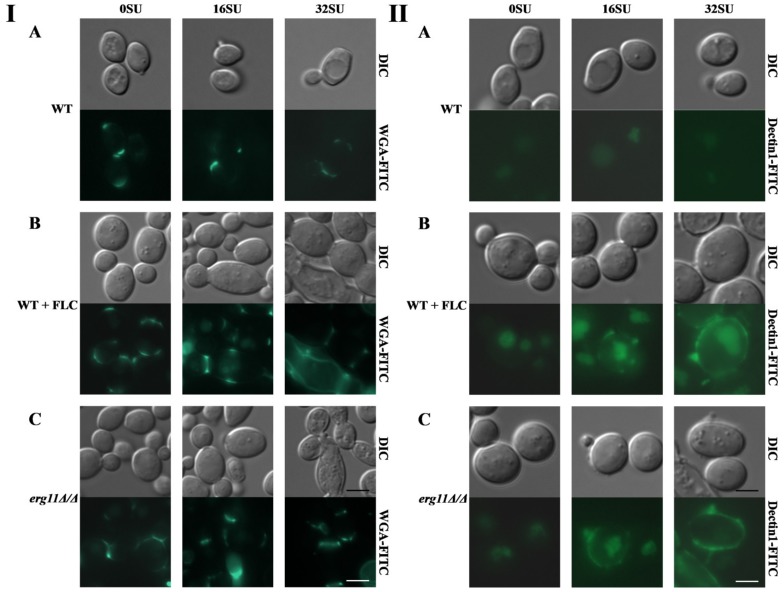
Unmasking chitin was detected by chitin staining with wheat germ agglutinin conjugated to fluorescein isothiocyanate (WGA-FITC) (panel (**I**)). Unmasking *β*-glucan was detected by *β*-glucan staining with Fc-hDectin-1 (FC-Dec1) and Alexa Fluor 448-conjugated anti-human IgG Fc antibodies (panel (**II**)) in *C. albicans* treated with 16 or 32 µg/mL SU. (**A**) *C. albicans* CAF2-1 (WT) strain; (**B**) *C. albicans* CAF2-1 (WT) strain simultaneously treated with 1 µg/mL fluconazole and (**C**) *C. albicans* KS028 (*erg11Δ/Δ*) mutant. Images are representative of at least three independent experiments. Scale bars are equal to 2.5 µm.

In both the *C. albicans erg11Δ/Δ* mutant and the WT strain treated with fluconazole, we observed a higher level of chitin unmasking in the cell wall than in *C. albicans* CAF 2-1 was observed. It seems that the addition of SU did not change the fluorescence of strains in these three examples but under the influence of SU we observed that some of the cells are elongated. Caspofungin by disrupting the cell wall structure in the septes separating parental and daughter cells causes the elongation of cells and their separation is impossible [17]. We have probably the same effect with SU (Figure 4A–C panel I). In the WT strain, we did not observe *β*-glucan exposure after staining. Treatment with SU did not result in visible staining (Figure 4A panel II). Similarly, unmasking *β*-glucan was not observed after WT cells were treated with fluconazole (Figure 4B panel II). In contrast, treatment of the WT strain with both fluconazole and SU resulted in the exposure of *β*-glucan, as illustrated by a strong fluorescent signal from Fc-hDectin-1 (Figure 4B panel II). Lack of ergosterol in the *erg11Δ/Δ* mutant caused slight exposure of *β*-glucan (Figure 4C panel II). The addition of SU to *C. albicans erg11Δ/Δ* significantly enhanced this effect. A strong fluorescent signal of *β*-glucan with Fc-hDectin1 staining was observed (Figure 4C panel II).

**Figure 5:**
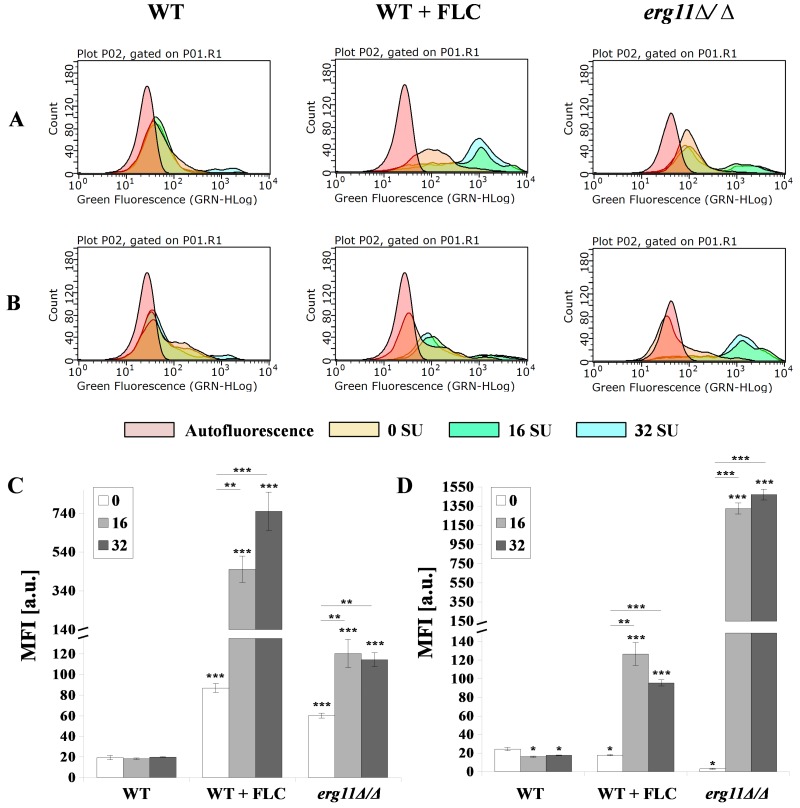
Exposure of (**A**,**C**) chitin and (**B**,**D**) *β*-glucan in *C. albicans* CAF2-1 (WT) or KS028 (*erg11Δ/Δ*) strains grown for 24h in YPD without and the addition of 16 or 32 µg/mL surfactin (0 SU, yellow; 16 SU, green and 32 SU, blue). The CAF2-1 strain was simultaneously treated with 1 µg/mL fluconazole (FLC) and SU. In each case, cells were fixed and stained with Fc-hDectin-1 and Alexa Fluor 448-conjugated anti-human IgG Fc antibodies or wheat germ agglutinin conjugated with FITC (WGA-FITC) and quantified by FACS (**A**,**B**). All results were compared to unstained CAF2-1 or KS028 cells (autofluorescence, red). Presented data are representative of three independent experiments. Median fluorescence intensities (MFIs) (**C**,**D**) were quantified for all three experiments. Autofluorescence of either CAF2-1 or KS028 unstained cells was subtracted. Statistical analyses were performed relative to control experiments using CAF2-1 untreated with SU (above bars) or in the case of WT + FLC and *erg11∆/∆* additionally between SU-treated cells (16SU or 32SU) or SU-nontreated (0SU) (above lines) (*, P < 0.05; **, P < 0.01; ***, P < 0.001).

We tested whether chitin and *β*-glucan are exposed after treatment with fluconazole and SU. These elements of the cell wall were stained with WGA-FITC (chitin) or FC-Dec1 (*β*-glucan) and the fluorescence intensity was quantified by flow cytometry. The addition of fluconazole to WT cells did not change the intensity of the fluorescent signal (Figure 5A,B—left and middle panel). The fluorescence of the *erg11Δ/Δ* mutant was significantly reduced (Figure 5A,B—right panel). The addition of fluconazole to the WT strain shifted the fluorescence to higher values for the chitin green fluorescence. Only a slight shift in *β*-glucan fluorescence was observed (Figure 5A,B—middle panel). The median fluorescence intensity (MFI) value indicates a 6.3-fold increase for chitin and stability for *β*-glucan (Figure 5C,D). Addition of fluconazole and SU to the WT strain shifted the green fluorescence of both to the right for chitin and *β*-glucan. MFI indicated an increase from 87 to 451.2 and 750.7 units for chitin. An increase from 17.7 to 126.5 and 95.6 units was observed for *β*-glucan depending on the concentration of SU (Figure 5A,B—left panel; Figure 5C,D). A similar but significantly enhanced effect of shifting green fluorescence to the right under the influence of SU was observed in the *erg11Δ/Δ* mutant (Figure 5A,B—middle and right panels). However, MFI values for chitin increased from 60.2 to 120.4 and 114.3 units depending on SU concentration. This effect was much smaller than the effect observed in the WT strain treated with fluconazole (Figure 5C,D). In contrast, MFI values for β-glucan increased from 3 to 1326 and 1470.9 at concentrations of 16 and 32 µg/mL SU, respectively (Figure 5C,D).

### 3.6. C. albicans erg11Δ/Δ Mutant Undergoes Increased Expression of Chitin and β-glucan Synthase Genes in Opposition to Surfactin Activity under these Conditions

**Figure 6:**
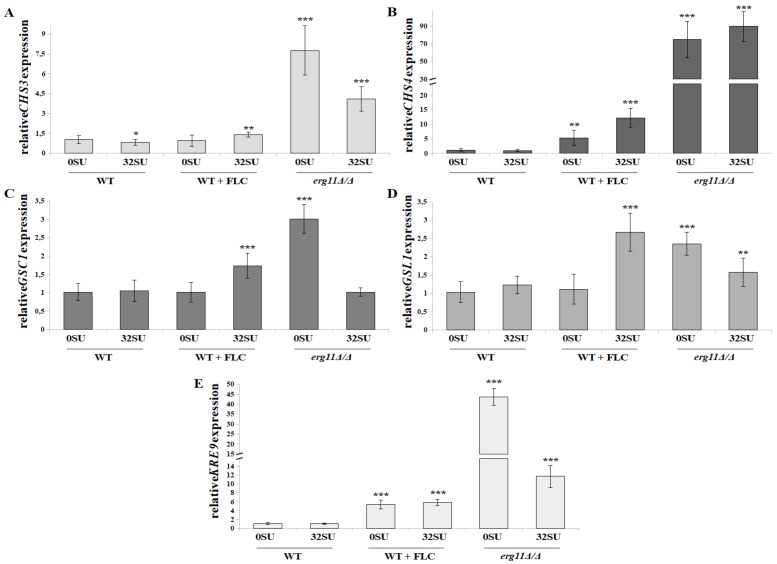
Relative *CHS3* (**A**), *CHS4* (**B**), *GSC1* (**C**), *GSL1* (**D**) or *KRE9* (**E**) gene expression in *C*. *albicans* treated with 32 µg/mL SU. The following cell types were treated under the following conditions: *C. albicans* CAF2-1 strain (WT), *C. albicans* CAF2-1 strain simultaneously treated with 1 µg/mL fluconazole (WT + FLC) and *C. albicans* KS028 strain (*erg11Δ/Δ* mutant). Gene expression levels are reported as means of 2^−∆∆CT^ values (n = 6) ± SD; normalized to 1 for untreated CAF2-1 strain (WT 0SU). Statistical analyses were performed by comparing expression under each condition to a untreated CAF2-1 strain (WT 0SU) (*, p < 0.05; **, p < 0.01; ***, p < 0.001).

Our results indicate an increased amount of chitin and *β*-glucan unmasking in the *C. albicans* cell wall. This occurs as a result of ergosterol depletion and/or SU treatment. These results prompted us to investigate the expression levels of genes encoding synthases of these cell wall elements. We investigated two genes encoding chitin synthases: *CHS3* and *CHS4.* In addition we investigated three gene encoding *β*-glucan synthases: *GSC1, GSL1* and *KRE9*. Chs3 and Chs4 proteins can propagate long chitin fibrils. CHS3p is also required for the formation of linkages between chitin and *β*-glucan. Previous studies have revealed that Chs3p is necessary for approximately 80% of chitin production in *C. albicans.* Studies have shown Chs4p is less involved in chitin formation, but it enhances the activity of Chs3 synthase [63]. We have observed that, in the *C. albicans erg11Δ/Δ* strain, *CSH4* and *CSH3* expression increased 7.8 and 74.8-fold, respectively (Figure 6A,B). Fluconazole treatment of the WT strain increased *CSH4* expression 5.3-fold, but not *CSH3* expression. In combination with SU, increased expression of *CSH3* was observed at the level of 1.4-fold. *CSH4* expression increased 2.2-fold (Figure 6A,B). SU exposure did not increase expression of the tested synthases in the WT strain. In the *C. albicans erg11Δ/Δ* strain, SU treatment resulted in a 1.9-fold decrease in *CSH3* expression and a 1.2-fold increase in *CSH4* expression (Figure 6A,B).

Chitin is covalently cross-linked with *β*(1,3)glucan and forms a primary scaffold that is responsible for structural integrity and cell wall shape [64]. In fungal cell walls, *β*-D-glucans are a (1→3)-linked glucose polymer with (1→6)-linked side chains [65]. Gsc1p and Gsl1p are putative subunits of the *β*-1,3-glucan synthase complex. The first enzyme acts as synthase, while the second acts as an activator [66]. *GSC1* gene disruption leads to an up to 20% decrease in cell wall *β*-glucan. In addition, there exists a direct correlation between the amount of *β*-glucan content and the amount of *GSC1* mRNA. These genes are targets for antifungal therapy, leading to inhibition of cell wall assembly factors [67,68]. *C. albicans* mutant with heterozygous disruption of the *KRE9* gene has been previously described. This mutant exhibits reduced levels of *β*-1,6-glucan in the cell wall whereas homozygous disruption leads to its total depletion. Thus, it has been proven that the *KRE9* gene encodes the enzyme responsible for *β*-glucan synthesis. The *KRE9* gene is a homologue of the *S. cerevisiae KRE9* gene which is involved in the synthesis of *β*-1,6-glucan molecules [69].

Our work has shown that, in a *C. albicans erg11Δ/Δ* mutant, the expression of genes encoding three tested *β*-glucan synthases (*GSC1, GSL1* and *KRE9*) is increased 3-, 2.4- and 43.7-fold, respectively compared to the WT strain (Figure 6C–E). SU does not affect the expression of these genes in the WT strain. However, in the *erg11Δ/Δ* mutant, it lowers *GSC1, GSL1* and *KRE9* gene expression 3-, 1.5- and 4- fold, respectively (Figure 6C–E). Fluconazole increases *KRE9* expression in a WT strain by 5.4-fold. In this case, SU does not affect gene expression (Figure 6E). The interaction of fluconazole and SU with WT cells caused increased expression of the *GSC1* and *GSL1* genes 1.7- and 2.5- fold respectively (Figure 6C,D).

### 3.7. Molecular Modeling Reveals that Surfactin Can Form Intermolecular Complexes with Chitin and β-glucan

**Figure 7:**
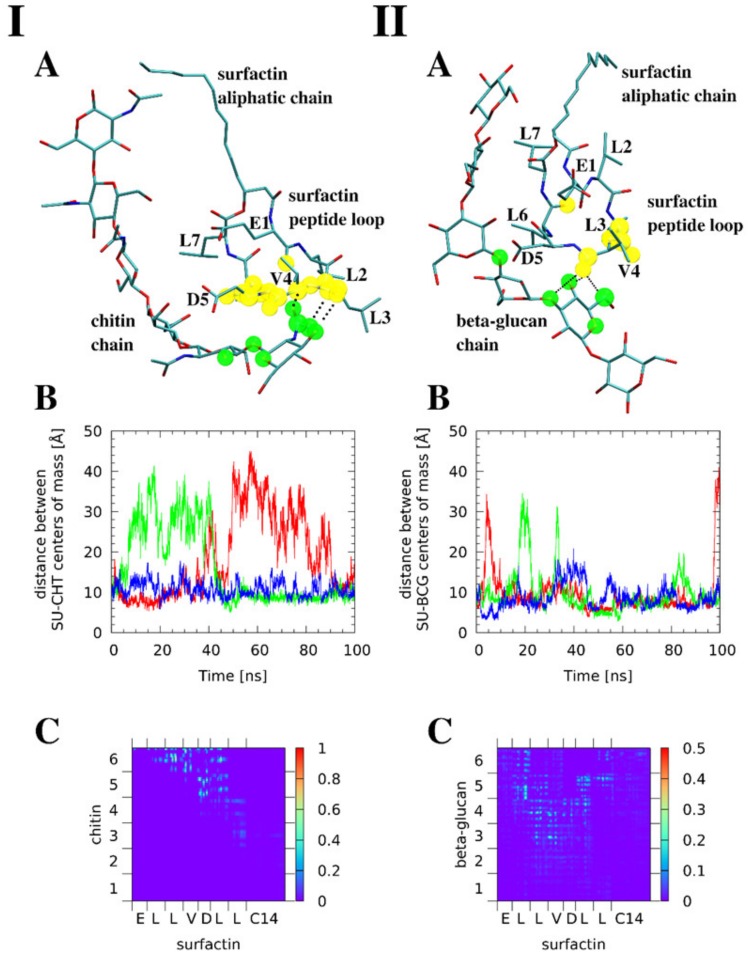
Molecular dynamic studies show that surfactin (SU) is able to form intermolecular complexes with chitin (**I**) and *β*-glucan (**II**). (**A**) Exemplary snapshots of the molecular dynamics runs; the atoms forming most stable close intermolecular contacts (<4 Å) are indicated by transparent spheres (green for the saccharide, yellow for SU). Color coding for atoms: cyan—carbon, blue—nitrogen, red—oxygen, while hydrogen atoms are not shown for clarity. Hydrogen bonds present in the snapshots are indicated by dotted lines. (**B**) Time evolution of distance between centers of mass of SU and the saccharide in three separate simulation runs. (**C**) Map of interatomic contacts between SU and the saccharide—the pixel color indicates the fraction of simulation time during which a particular interatomic contact existed.

Molecular dynamics studies were carried out using a classical force field for 1:1 SU-chitin and SU-*β*-glucan noncovalent complexes. The C14 SU from *B. subtilis* was considered as a model. First, the chitin complexes will be discussed. A model of chitin chain, linear hexamer of *N*-acetylglucosamine was found to bind to a part of the SU peptide loop (Figure 7A panel I). Three separate 100 ns runs of classical molecular dynamics were studied. These use the same initial structures, but diverse initial velocities and thermostats, resulting in a quick divergence of simulation trajectories. One of the simulations gave a continuously stable complex, while the remaining two complexes dissociated and bound again. These complexes were bound approximately half of the simulation time (Figure 7B panel I). This was determined using the time evolution of distance between the centers of mass of SU and chitin. The MD trajectory analysis shows that the SU peptide loop is somewhat rigid, while the aliphatic chain is conformationally labile. The chitin chain remains linear while the sugar residues oscillate around the chain axis.

A map of intermolecular contacts was prepared (Figure 7C panel I) to indicate the fraction of time that a given atom pair from SU and chitin were less than 4 Å apart. We assumed this 4 Å cutoff, which is larger than conventionally used limits of hydrogen bonding (3.0 to 3.2 Å), due to the dynamic nature of the complexes in addition to the limitations of classical force fields when reproducing noncovalent interactions. The panels I A,C of Figure 7 show that the interactions exist mostly between SU residues L3, V4, D5, L6 and the sugar units 5 and 6 in the hexameric chitin model. For each SU residue shown on the X axis of Panel C, the sequence of plotted atoms is: backbone (N, C, O) and side-chain (Cα, Cβ). The hydrogen atoms were omitted from the contact analysis. The interactions are dynamical, as they are being continually formed and broken. The most important contacts exist for about 80% of the simulation time.

Interestingly, as indicated in Panel I A of Figure 7, the contact atoms are located in the backbone, not the sidechains, of the SU peptide loop. This accounts for spatial proximity the hydroxyl functions of the middle part of the chitin chain and the carboxyl sidechains of the E1 or D5 residues. This potentially enables the formation of ester bonds between these groups. Such interaction involves covalent bonding of the two moieties, thus it is outside the scope of this molecular dynamics study. The results of the simulations of SU-*β*-glucan complex (Figure 7 II) revealed some important differences between the affinity of SU toward *β*-glucan and chitin. The SU-*β*-glucan complexes rarely dissociate. When dissociation is observed, it occurs for a much shorter period than the SU-chitin systems (Panel II B of Figure 7). They are stable for over 90% of the simulation time. This, however, does not mean that the intermolecular contacts are more stable. Panel II C of Figure 7 reveals that the most stable contacts persist for less than 40% of the simulation time. They are formed by the SU residues L2, L3, V4, D5, L6 and the sugar units 3 to 5 of the hexameric *β*-glucan model. The discrepancy between the overall better stability and loss of persistence of particular contacts within the SU-*β*-glucan complexes can be explained by their stronger dynamics. Contacts are constantly broken and re-established possibly changing the donor-acceptor pairs.

## 4. Discussion

*C. albicans* has developed several defense mechanisms to survive environmental stress produced by the host immune system as well as antifungal agents. The resistance of this fungus is most often facilitated by changes in the structure and functioning of the cell wall and the plasma membrane. Antifungal compounds currently used function to block membrane (azoles) or cell wall (echinocandins) functions separately. However, this tactic is becoming less and less effective [70,71]. The most commonly used azole drug is fluconazole and the most commonly used echinocandin drug is caspofungin. Caspofungin (lipopeptide) induces expression of several genes encoding cell wall’s proteins [72,73] and triggers cell wall salvage mechanisms [74]. Another lipopeptide known for its antibacterial properties, surfactin (SU), did not show significant activity against the viability of *C. albicans* in our previous studies [15]. In contrast, several other teams have shown SU activity against fungal plant pathogens. One of the effects of SU is change in the shape of the fungal cells, likely due to changes in the cell wall [75,76,77].

Unexpectedly, we have observed high sensitivity to SU in an ergosterol-deficient *C. albicans* mutant (*erg11Δ/Δ*) (Figure 1). Combinations of triazoles (fluconazole and itraconazole) with SU significantly reduced the value of concentration leading to ≥50 and ≥90% growth inhibition for *C. albicans* CAF2-1 strain (WT). Combinations of imidazoles with SU lowered ≥90% growth inhibition concentration values but did not give the above effect in case of ≥50% growth inhibition (Table 1). This data is especially promising taking into account previous reports on cytotoxic concentrations of SU towards human and animal cell lines [78,79]. They were in a range of 38–80 µg/mL, which is below the SU concentrations used in combinations with azoles against *C. albicans* (Table 1). Triazoles and imidazoles block cytochrome P450 isozymes with different selectivities [80,81]. These drugs affect ergosterol synthesis, lipid composition and the function of cell membranes with varying intensity [82]. Azole also affects the activity of chitin synthases, resulting in an irregular distribution of chitin in the cell wall [83]. Our results suggest that lack of ergosterol, or total depletion by blocking its synthesis pathway by triazoles, causes adequate changes in the cell wall. The cell wall then becomes susceptible to the influence of SU. The above hypothesis has been confirmed by intensive membrane permeabilization in the *erg11Δ/Δ* mutant under the influence of SU and in *C. albicans* WT in the presence of fluconazole and SU (Figure 2).

Dupont et al. [52] proved that the composition of sterols in the plasma membrane governs its mechanical behavior. Dupont’s team showed increased susceptibility to the osmotic fluctuation of a yeast *S. cerevisiae* mutant with impaired ergosterol synthesis. The hypersensitivity of the mutant was linked to cell volume variation and PM permeabilization. Moreover, ergosterol supplementation significantly increased mutant survival and resistance to osmotic shock [52]. In our investigations, a *C. albicans erg11Δ/Δ* mutant and the *C. albicans* WT strain show cell deformation after fluconazole treatment. We observed dimples in the cells were clearly deepened due to the action of SU (Figure 3A). We also found localized accumulation of chitin in the cell wall of *C. albicans* lacking ergosterol and in a *C. albicans* WT strain treated with fluconazole. We did not observe an increase in the accumulation of chitin in the cells treated with fluconazole and SU. This was in contrast to the cells of a mutant without ergosterol after adding SU (Figure 3C). These results correlate with our studies on the expression of genes encoding selected chitin synthases. We did not observe increased expression of these genes under the influence of SU (Figure 6A,B). These results suggest a different mechanism of SU antifungal activity compared to caspofungin.

The cell wall polysaccharides *β*-glucan and chitin are responsible for structural integrity and shape of the cell wall. *β*-glucan is linked with mannoproteins that form the outermost layer of the cell wall and these two elements of the cell wall are mainly responsible for host-pathogen interactions [19,64]. *β*-glucan is an essential cell wall component targeted by fungicidal antibodies and immune receptors. Dectin-1, the *β*-glucan receptor, also recognizes fungi and mediates the immune system’s proinflammatory response [84,85]. *β*-glucan must be unmasked by the removal of mannoproteins in order to activate the immune system. Thus, *C. albicans* often hide from the immune system’s pro-inflammatory response by masking *β*-glucan [62]. For these reasons, it is essential to treat with drugs that expose *β*-glucan to the immune system. Wheeler and Fink [62] showed that caspofungin in subinhibitory concentrations exposes the normally-masked *β*-glucan. This is an additional activity of this drug, as it also blocks chitin synthesis [86]. Caspofungin is thus a dual-action antifungal drug. *C. albicans* cells defend against caspofungin by stimulating chitin synthesis [87].

When the microscopic observations in this study are taken into consideration, the effect of unmasking *β*-glucan in the WT strain through the use of SU and fluconazole is observed (Figure 4 p. II B). It seems that the combination of these compounds did not increase chitin unmasking by fluconazole (Figure 4 p. I B). When the *erg11Δ/Δ* mutant is treated with SU we did not observe an increase in chitin unmasking (Figure 4 p. I C). This observation was in contrast to intensive *β*-glucan unmasking (Figure 4 p. II C). The above observations, in most cases, were confirmed by the quantitative results from FACS studies (Figure 5). Also, we observed that when fluconazole and SU were used, the amount of unmasked chitin measured by MFI increased in the range 6- to 8-fold depending on the SU concentration (Figure 5C). This result suggests that SU participates in chitin unmasking.

SU treatment caused an *erg11Δ/Δ* mutant to reduce expression of the *CHS3* gene which is estimated to account for 80% of chitin production in the cell wall [63]. SU treatment also lowered expression of genes encoding *β*-glucan (Figure 6). Based on these results, it seems that only very low or absent ergosterol induces a reaction involving the accumulation of chitin. SU does not enhance chitin accumulation. When there is a complete lack of ergosterol in the cell, SU inhibited chitin and *β*-glucan synthesis. SU treatment also exposed both components of the cell wall to the immune system. To clarify whether SU can bind to *β*-glucan and chitin, we conducted in silico investigations. Coupling studies showed that the overall stability of the complexes is higher for SU-*β*-glucan. We tentatively assigned these interactions as stronger than the SU-chitin noncovalent complexes. Interestingly, we observed that the protein-polysaccharide contacts were formed by the protein loop of SU while the hydrophobic aliphatic chain remains free (Figure 7).

In this work, we showed the potential use of SU as an antifungal drug with a different mechanism of action than another lipopeptide, caspofungin. Although the combination of caspofungin with azoles demonstrated in vitro activity against pathogenic fungi, clinical studies provided uncertain data whether this combination was active in vivo [88]. This indicates that the next stage of our research should be testing the effect of SU on animal infection models. Such research would be especially promising because numerous reports are currently available concerning low cytotoxic properties of SU in vitro [78,79]. According to our results, SU binds to *β*-glucan and binds with a weaker affinity to chitin, revealing these elements of the *C. albicans* cell wall to the immune system. In order to unmask these elements, changes in the plasma membrane are necessary. These changes require a reduced amount or lack of ergosterol. Hence, the presence of fluconazole, which depletes or removes ergosterol, is beneficial. The cell resists killing by SU by reducing the expression of chitin-encoding genes. This is a reversible process that protects against azoles.

## 5. Conclusions

*Candida albicans* is an opportunistic pathogen that resides in the human microflora. Systemic *C. albicans* infections are often fatal in immunocompromised individuals. New antifungal therapeutics are needed due to a limited number of effective treatment compounds. Biosurfactants like lipopeptides are promising therapeutic compounds with diverse biological activities. Surfactins are lipopeptide biosurfactants consisting of a seven amino acid peptide loop attached to a hydrophobic fatty acid.

This study shows that surfactin enhances the antifungal activity of fluconazole. Fluconazole lowers the ergosterol concentration in the plasma membrane, thereby modifying the structure of the cell wall. Fluconazole enables surfactin to expose *β*-glucan to the host immune system. Surfactin can form complexes with *β*-glucan and chitin, leading to complex accumulation and further cell wall and plasma membrane modification. Modulating interactions between the plasma membrane and the cell wall in pathogenic fungi is a viable potential antifungal strategy.

## Figures and Tables

**Table 1:** Concentrations (µg/mL) of triazoles (fluconazole and itraconazle) or imidazoles (ketoconazole, clotrimazol, miconazole) leading to either ≥ 50 or ≥ 90% growth inhibition (g.i.) of *C. albicans* CAF2-1 strain. These values were given alone or in combination with surfactin (SU) (µg/mL) towards *C. albicans* CAF2-1 strain. Fractional inhibitory concentration indexes (FICIs) were included in bracelets.

Azole	≥50% g.i.	≥50% g.i.	≥50% g.i.	≥90% g.i.	≥90% g.i.	≥90% g.i.
		16 µg/mL SU	32 µg/mL SU		16 µg/mL SU	32 µg/mL SU
**Triazoles**						
**Fluconazole**	2	0.5	0.5	>256	1	0.5
		(FICI = 0.258)	(FICI = 0.258)		(FICI < 0.012)	(FICI < 0.010)
**Itraconazole**	0.0313	0.0078	0.0039	>8	0.0156	0.0078
		(FICI = 0.257)	(FICI = 0.132)		(FICI < 0.010)	(FICI < 0.009)
**Imidazoles**						
**Ketoconazole**	0.0039	0.0039	0.0039	4	0.0078	0.0078
		(FICI = 1.008)	(FICI = 1.008)		(FICI = 0.010)	(FICI = 0.010)
**Clotrimazole**	0.0156	0.0156	0.0156	1	0.0313	0.0313
		(FICI = 1.008)	(FICI = 1.008)		(FICI = 0.039)	(FICI = 0.039)
**Miconazole**	0.0156	0.0156	0.0156	2	0.0313	0.0156
		(FICI = 1.008)	(FICI = 1.008)		(FICI = 0.023)	(FICI = 0.016)
